# Kidney Agenesis and Müllerian Duct Anomalies: A Report of Two Cases and Literature Review

**DOI:** 10.15388/Amed.2025.32.1.7

**Published:** 2025-02-18

**Authors:** Kamilė Donielaitė-Anisė, Rytis Marozas, Žana Bumbulienė, Augustina Jankauskienė

**Affiliations:** 1 Vilnius University Hospital Santaros klinikos, Vilnius, Lithuania Faculty of Medicine, Vilnius University, Vilnius, Lithuania E-mail: ORCID ID; 2 Radiology and Nuclear Medicine Center, Vilnius University Hospital Santaros Klinikos, Vilnius, Lithuania Faculty of Medicine, Vilnius University, Vilnius, Lithuania E-mail:; 3 Clinic of Obstetrics and Gynecology, Vilnius University Hospital Santaros Klinikos, Vilnius, Lithuania Faculty of Medicine, Vilnius University, Vilnius, Lithuania E-mail: ORCID ID; 4 Centre of Pediatrics, Vilnius University Hospital Santaros Klinikos, Vilnius, Lithuania Faculty of Medicine, Vilnius University, Vilnius, Lithuania E-mail: ORCID ID

**Keywords:** Kidney agenesis, renal agenesis, Müllerian duct anomalies, OHVIRA, Herlyn-Werner-Wunderlich syndrome, inkstų agenezė, Miulerio latakų anomalijos, *Herlyn-Werner-Wunderlich* sindromas

## Abstract

**Background:**

The association between urinary tract anomalies and *Müllerian duct anomalies* (MDA) is well-known, due to their shared embryonic origin. Disruptions in early development can significantly affect both the kidney and reproductive systems. This article presents two cases illustrating the coexistence of kidney agenesis and MDA in girls, followed by a literature review.

**Materials and Methods:**

A literature search was conducted on *PubMed*, focusing on publications from 2000 to 2024 by using keywords: ‘kidney agenesis’, ‘renal agenesis’, ‘Müllerian duct anomalies’, ‘OHVIRA’ (obstructed hemivagina and ipsilateral renal anomaly), and ‘Herlyn-Werner-Wunderlich syndrome’. The PRISMA guidelines were followed for the study selection. Additionally, two cases managed at Vilnius University Hospital Santaros Klinikos between 2022 and 2024 are presented.

**Results:**

The literature search yielded 32 articles encompassing data on 43 girls with an average age of 11.8 years. In 54% of the cases, the diagnosis of kidney agenesis was concurrent with identifying MDA. In other cases, kidney anomalies were detected earlier, including 6 cases identified prenatally. Type III MDA, as classified by the *American Fertility Society*, was the most common variety. Premenarche diagnosis of MDA was made in 11.9% of the cases. In more than half of the cases, MDA was identified due to complaints necessitating consultations, mostly leading to urgent surgical interventions. At our hospital, a 9-year-old and a 14-year-old were diagnosed with Herlyn-Werner-Wunderlich syndrome. Kidney agenesis was diagnosed prior to MDA in both cases. For the 9-year-old girl, MDA was found incidentally on ultrasound, while the other required consultation and an urgent surgery due to symptoms.

**Conclusions:**

Unilateral kidney agenesis frequently co-occurs with Müllerian duct anomalies, highlighting the need for comprehensive evaluations in affected patients. An early diagnosis and management of MDA are crucial to prevent complications. An increased clinical awareness and further research are necessary to enhance early detection and patient outcomes.

## Abstract (it)

**Įvadas:**

Inkstų ir Miulerio latakų anomalijos dažnai pasireiškia kartu dėl jų bendros embrioninės kilmės. Vystymosi sutrikimai ankstyvojo nėštumo metu gali reikšmingai paveikti tiek inkstų, tiek reprodukcinę sistemas. Šiame straipsnyje pateikiama literatūros apžvalga ir du atvejai, iliustruojantys inkstų agenezės ir Miulerio latakų anomalijas.

**Medžiaga ir metodai:**

Literatūros paieška atlikta *PubMed* sistemoje, analizuoti straipsniai publikuoti nuo 2000 iki 2024 metų. Naudoti raktažodžiai: inkstų agenezė, Miulerio latakų anomalijos, *Herlyn-Werner-Wunderlich* sindromas, OHVIRA (*obstrukcinė hemivagina ir ipsilateralinė inkstų anomalija*). Tyrimų atrankai buvo naudojamos PRISMA gairės. Taip pat pateikiami du atvejai, pacientės gydytos Vilniaus universiteto ligoninės Santaros klinikose.

**Rezultatai:**

Rasti 32 straipsniai, juose buvo duomenų apie 43 mergaites, kurių vidutinis amžius – 11,8 metų. Inkstų agenezės diagnozė 54 % atvejų buvo nustatyta kartu su Miulerio latakų anomalijomis, kitais atvejais inkstų anomalijos buvo aptiktos anksčiau, 6 atvejais – prenataliai. Dažniausiai nustatyta III tipo Miulerio latakų anomalija (pagal Amerikos vaisingumo draugijos klasifikaciją). Miulerio latakų anomalijos diagnozė prieš menarchę buvo nustatyta 11,9 % atvejų. Daugiau nei pusę atvejų Miulerio latakų anomalija nustatyta mergaitėms, kreipiantis turint simptomų, dažniausiai reikalaujančių skubios chirurginės intervencijos. Mūsų ligoninėje gydytos 9 ir 14 metų mergaitės, kurioms buvo diagnozuotas *Herlyn-Werner-Wunderlich* sindromas. Abiem atvejais inkstų agenezė buvo nustatyta anksčiau nei gimdos. Gimdos anomalija 9 metų mergaitei buvo nustatyta atsitiktinai ultragarsinio tyrimo metu, kitai mergaitei kreipiantis dėl skundų, kai reikėjo skubios chirurginės intervencijos.

**Išvados:**

Vienpusė inkstų agenezė dažnai būna kartu su Miulerio latakų anomalijomis, todėl svarbu nustačius vieną anomaliją atlikti išsamų pacienčių ištyrimą. Siekiant išvengti komplikacijų, svarbu anksti diagnozuoti Miulerio latakų anomalijas. Klinikinis budrumas ir tolesni moksliniai tyrimai yra svarbūs siekiant pagerinti ankstyvą anomalijų diagnozavimą ir gerinti pacienčių gydymo baigtis.

## Introduction

The association between the urinary tract and *Müllerian duct anomalies* (MDA) has been acknowledged for a relatively long time. This association stems from their shared embryological origin within the urogenital ridge, where disruptions during early development can profoundly impact the kidney and reproductive systems [1]. Furthermore, both anomalies are often diagnosed later in life, sometimes not until puberty or adulthood, potentially leading to significant clinical complications [2]. An early diagnosis is crucial for improved patient outcomes, as well as for minimising the risk of long-term health issues [3]. Given this significant association, a diagnosis of either kidney agenesis or a Müllerian duct anomaly necessitates a comprehensive evaluation for the other to ensure timely intervention and the optimal patient care.

Müllerian duct anomalies are commonly classified according to two major systems. The *American Society for Reproductive Medicine* (ASRM) categorises MDAs primarily based on the uterine morphology into seven classes, including hypoplasia/agenesis, unicornuate, didelphys, bicornuate, septate, arcuate, and *diethylstilbestrol* (DES)-related anomalies [4]. The *European Society of Human Reproduction and Embryology*/*European Society for Gynecologic Endoscopy* (ESHRE/ESGE) classification system encompasses uterine, cervical, and vaginal anomalies, and organises them into six primary classes with further subdivisions based on associated anomalies [5]. Notably, many case reports in the literature describe Müllerian anomalies without any formal classification, especially in cases of uterus didelphys.

This article presents two cases illustrating the coexistence of kidney agenesis and MDA in girls, followed by a literature review.

## Methods

**Figure 1:**
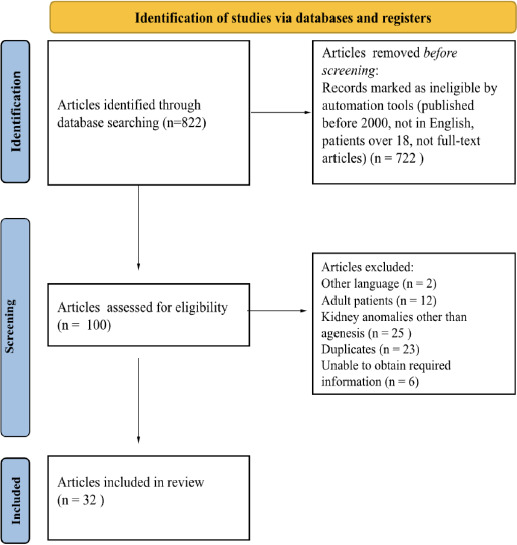
Flowchart of selected articles

The literature search was performed on the *PubMed* database by using the keywords: ‘kidney agenesis’, ‘renal agenesis’, ‘Müllerian duct anomalies’, ‘OHVIRA’, ‘Herlyn-Werner-Wunderlich syndrome’ (HWWS), while focusing on data published from 2000 to 2024. Figure 1 represents the study selection process, following the guidelines of the *Preferred Reporting Items for Systematic Reviews and Meta-Analyses* (PRISMA-P Statement).

In addition, two cases of Herlyn-Werner-Wunderlich syndrome, also known as OHVIRA (obstructed hemivagina and ipsilateral renal anomaly), managed at Vilnius University Hospital Santaros Klinikos between 2022 and 2024, are presented.

## Results

Our literature search yielded a final selection of 32 articles, including 26 case reports, 5 case series, and 1 research article. The selected articles provided data on a total of 43 girls, with a mean age of 11.8 ± 4.8 years. Kidney agenesis and Müllerian duct anomalies often present together, with simultaneous diagnoses occurring in 54% of cases. In other cases, kidney agenesis was diagnosed prior to MDA. Prenatal diagnosis of kidney agenesis was made in only 13.9% of the cases. In one of those cases, both kidney agenesis and Type III MDA were diagnosed. Seven girls were diagnosed with additional congenital disorders: 2 with VACTERL association, 2 with scoliosis, 1 with teratoma, 1 with multiple anomalies, and 1 with Prader-Willi syndrome. The most prevalent Müllerian duct anomaly was Type III (didelphys uterus), found in 74.4% of the cases. Only a small percentage (11.9%) of MDAs were diagnosed before the onset of menstruation. Alarmingly, 82.8% of the girls in the studies required medical consultation for their symptoms, mostly leading to urgent surgical interventions. Table 1 presents data on the authors, publication year, article type, population age, first detected pathology, associated malformations, MDA type, premenarche diagnosis, the circumstances of MDA detection, and the need for urgent surgery.

**Table 1:** Characteristics of studies included in literature review

Author, publication year	Article type	Patientage (years)	First detected pathology (kidney, MDA or both)	Other congenital disorders	MDA type (ASRM classification)	Premenarche diagnosis	Circumstances	Urgent surgery
A. Chapagain et al. [6], 2022	Case report	10	Both	No	III	No	Due to complaints	Yes
K. Karimbayev et al. [7], 2018	Case report	14	Kidney agenesis	No	Ie	Other*	Incidentally, during USG	N.i.
L. Li et al. [8], 2021	Research (8 patients)	13.2	N.i.	N.i.	III (5 cases) IVb (1 case) Va (2 cases)	No	N.i.	N.i.
W. Horst et al. [9], 2021	Case report	14	Kidney agenesis	No	III	No	Due to complaints	Yes
D. Lamichhane et al. [10], 2023	Case report	14	Both	No	III	No	Due to complaints	Yes
Z. Li et al. [11], 2014	Case report	12	Both	Scoliosis	III	No	Incidentally, during USG	No
J. Kang et al. [12], 2018	Case report	11	Both	VACTERL association	IVb+Ia	No	Due to complaints	Yes
G. Morabito et al. [13], 2020	Case report	4	Both	No	Ie	Yes	Incidentally, during USG	No
G. Niu et al. [14], 2020	Case report	11	Kidney agenesis (prenatal)	No	IVa	No	Due to complaints	Yes
A. Luther et al. [15], 2011	Case report	13	Both	No	IIb	No	Due to complaints	Yes
R. Del Vescovo et al. [16], 2012	Case series (3 patients)	14.7	Both (3 cases)	No	III (2 cases) IIb (1 case)	No (3 cases)	Due to complaints (3 cases)	N.i.
R. Obeidat et al. [17], 2019	Case report	17	Kidney agenesis	VACTERL association	III	No	Due to complaints	Yes
A. Samanta et al. [18], 2022	Case report	11	Kidney agenesis	No	III	No	Due to complaints	Yes
D. Cox et al. [19], 2012	Case report	17	Both	No	III	No	Due to complaints	Yes
M. Jhirwal et al. [20], 2021	Case report	14	Both	Scoliosis	III	No	Due to complaints	Yes
A. Coskun et al. [21], 2008	Case report	16	Both	Multiple malformations	III	No	Due to complaints	Yes
T. Wu et al. [22], 2012	Case report	0	Kidney agenesis (prenatal)	No	III	Yes	Due to complaints	Yes
A. Aveiro et al. [23], 2011	Case report	13	Kidney agenesis (prenatal)	No	III	No	Due to complaints	Yes
V. Fontana et al. [24], 2024	Case series (2 patients)	13	Kidney agenesis (1 prenatal)	No	IVa, Va	No (2 cases)	Due to complaints (1case), incidentally, during USG (1 case)	Yes (1 case)
M. Kozlowski et al. [25], 2020	Case series (2 patients)	15	Both (1 case), kidney agenesis (1 case)	No (2 cases)	III (2 cases)	No (2 cases)	Due to complaints (2 cases)	Yes (2 cases)
D. Nishu et al [26], 2019	Case report	15	Both	No	III	No	Due to complaints	Yes
D. Albulescu et al. [27], 2018	Case series (1/2 patients)**	14	Both	No	III	No	Due to complaints	No
B. Fraga et al. [28], 2015	Case report	0	Kidney agenesis (prenatal)	Prader-Willi syndrome, ventricular septal defect	III	Yes	Incidentally, during USG	No
S. Kumar et al. [29], 2015	Case report	14	Both	No	III	No	Due to complaints	Yes
A. Daoub et al. [30], 2014	Case report	12	Kidney agenesis	No	III	No	Due to complaints	Yes
S. Kim et al. [31], 2023	Case report	0	Both (prenatal)	No	III	Yes	Incidentally, during USG	Yes
B. Asha et al. [32], 2007	Case report	17	Both	No	III	No	Due to complaints	Yes
G. Kudela et al. [33], 2019	Case report	13	Kidney agenesis	No	III	No	Due to complaints	Yes
K. Mitani et al. [34], 2021	Case report	0.75	Both	Teratoma	III	Yes	Due to complaints	Yes
D. Shah et al. [35], 2011	Case report	12	Kidney agenesis	No	III	No	Due to complaints	Yes
M. Aranke et al. [36], 2018	Case report	13	Kidney agenesis	No	III	No	Due to complaints	Yes
K. Imaeda et al. [37], 2021	Case series (¼ patients)**	12	N.i.	No	III	No	Due to complaints	Yes

* The patient did not have a womb; ** Other patients were adults, so not included; N.i. – no information; US – ultrasound

**Figure 2:**
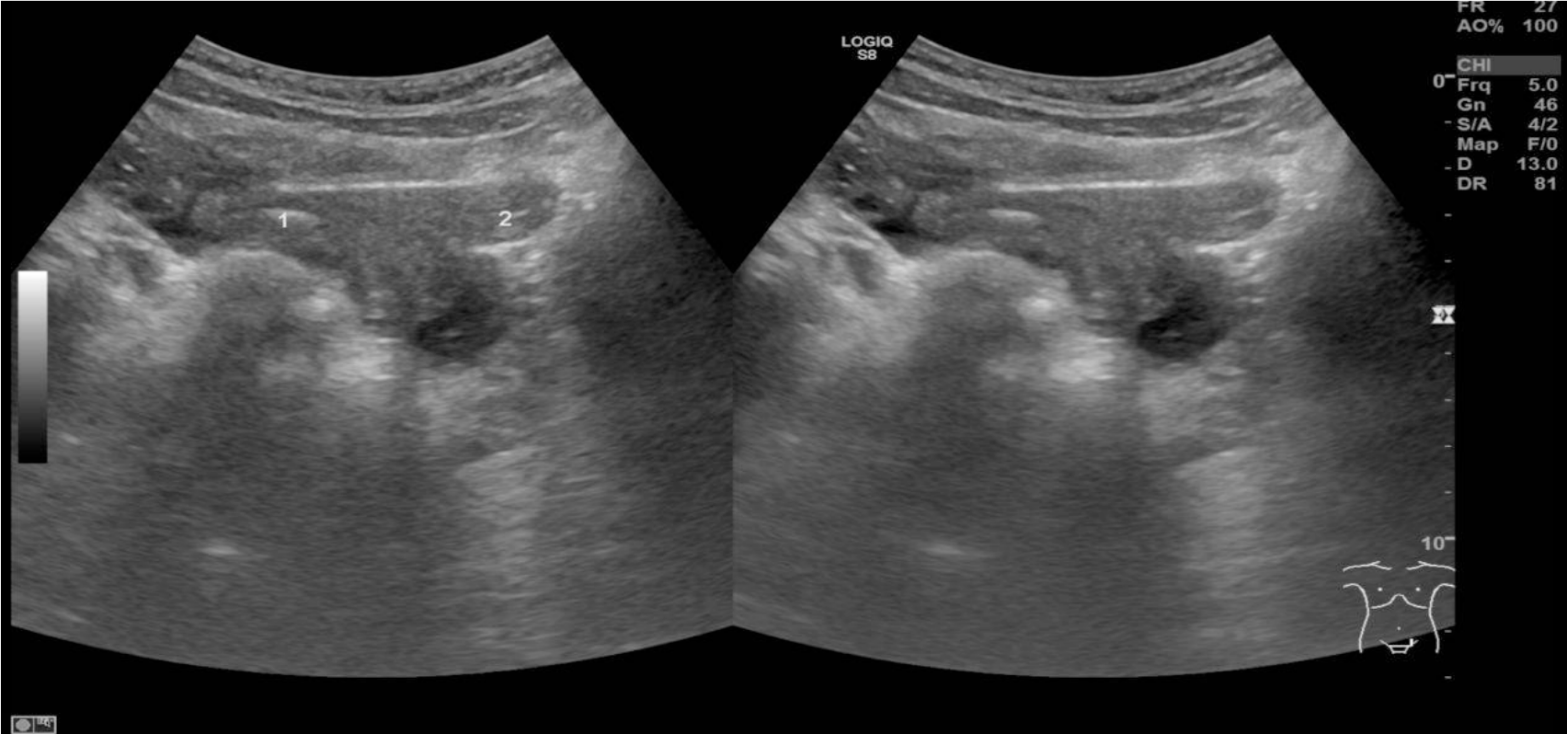
Ultrasound images of double corpuses of uterus with two separate linings of endometrium.

**Figure 3:**
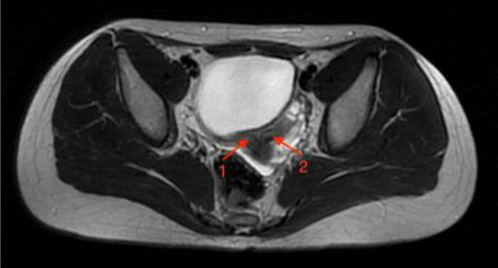
T2 weighted MR axial image of the pelvic region demonstrates double corpuses of uterus (arrows 1 and 2) with two separate linings of endometrium and two normal ovaries

We present two cases managed at our hospital:

Case 1: A 9-year-old female was evaluated during a prophylactic visit following the incidental finding of a left kidney agenesis one year prior to this evaluation. No complaints were reported at the time of assessment. An ultrasound session revealed a potential uterine anomaly (Figure 2), and an MRI was performed, demonstrating double corpuses of the uterus with two separate linings of the endometrium, and two normal ovaries (Figure 3), as well as two separate uterine cervices with the cystic structure on the left side. The diagnosis of Herlyn-Werner-Wunderlich syndrome was made. A gynecologist consultation was performed, and a decision was made to postpone the surgical treatment until menarche.

**Figure 4:**
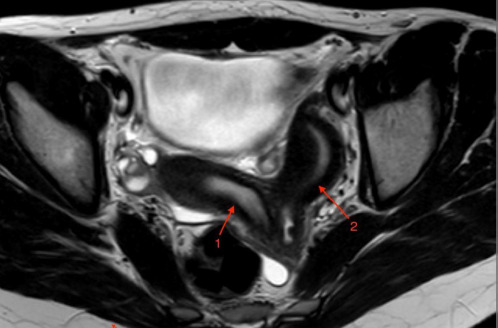
T2 weighted MR axial image demonstrates two separate uteruses (arrows 1 and 2) with two separate vaginas – uterus didelphys

Case 2: A 14-year-old girl presented to a gynecologist with severe menstrual pain and irregular bleeding. She was being monitored by a pediatric nephrologist due to left kidney agenesis. Her gynecological evaluation revealed an abnormal uterine structure, and an MRI demonstrated two separate uteruses with two separate vaginas – uterus didelphys (Figure 4), hydrocolpos on the left side, and a thin communication with the right normal vagina. The diagnosis of Herlyn-Werner-Wunderlich syndrome was made.

The summarised data of the patients are presented in Table 2.

**Table 2:** Characteristics of two cases with kidney agenesis and Müllerian duct anomaly

	Case 1	Case 2
Kidney anomaly	Left kidney agenesis	Left kidney agenesis
Reproductive organs anomaly (classification by the American Fertility Society)	Type III	Type III
Pre-menarche diagnosis	Yes	No
Circumstances	Incidentally, during ultrasound examination	Gynecologist consulted the girl because of complaints
Treatment strategy	Surgical treatment after menarche	Urgent surgery due to complaints

## Literature Review and Discussion

Kidney and Müllerian duct anomalies are known to co-occur due to their shared embryonic origin in the urogenital ridge, developing simultaneously in early gestation [1]. Kidney development spans from the 3^rd^ to the 36^th^ week, while the Müllerian system forms between the 5^th^ and the 15^th^ weeks [2,39,40]. Unilateral kidney agenesis, affecting approximately 1 in 2,000 births [41], is frequently asymptomatic and is diagnosed later in life [2]. MDA, affecting about 5.5% of the female population, exhibits a wide spectrum of severity, ranging from minor septations to complete organ absence [42]. The prevalence of Herlyn-Werner-Wunderlich syndrome is estimated to range from 0.1% to 3% [23].

While the exact causes of both conditions remain unclear, a complex interplay of genetic and environmental factors is suspected. Kidney agenesis results from a developmental failure of the ureteric bud and the metanephric mesenchyme during early organogenesis. While it often occurs sporadically, it can also be part of syndromic conditions involving broader developmental defects. Mutations in several genes, including **RET** (10q11.2), **BMP4** (14q22-q23), **FRAS1** (4q21.21), **FREM1** (9p22.3), and **UPK3A** (22q13.31), have been implicated in unilateral kidney agenesis [41]. Kidney agenesis frequently co-occurs with other urological anomalies (30%), and may also involve defects in the cardiovascular, gastrointestinal, and skeletal systems [43]. Notably, over one-third of kidney agenesis cases present with concurrent Müllerian anomalies [38]. MDAs result from a disruption in one of three developmental phases: duct formation, fusion, or septal reabsorption [44]. Although the exact genetic etiology of HWWS is unknown, there might be an association with the genes implicated in unilateral kidney agenesis, such as RET and FRAS1 [45]. Further studies are required to elucidate these genetic associations along with the underlying molecular mechanisms.

In modern times, the most widely used classification systems for Müllerian duct anomalies are those of the *American Society for Reproductive Medicine* (previously the *American Fertility Society*), as well as the *European Society of Human Reproduction and Embryology* and the *European Society for Gynecologic Endoscopy*. In 1988, ASRM published its initial classification system of Müllerian duct anomalies. This classification primarily focuses on uterine anomalies, with limited references to the cervix and vagina. The ASRM classification organizes anomalies by the major uterine anatomical types, with some classes including subtypes that describe specific variants: Class I – hypoplasia/agenesis (subtypes: a – vaginal, b – cervical, c – fundal, d – tubal, e – combined), Class II – unicornuate uterus (subtypes: a – communicating, b – non-communicating, c – no cavity, d – no horn), Class III – didelphys uterus, Class IV – bicornuate uterus (subtypes: a – complete, b – partial), Class V – septate uterus (subtypes: a – complete, b – partial), Class VI – arcuate uterus, and Class VII – diethylstilbestrol-related anomalies [4]. In 2013, ESHRE/ESGE published a comprehensive classification system for congenital anomalies of the female genital tract, which encompassed not only uterine anomalies but also cervical and vaginal anomalies. The ESHRE/ESGE classification system divides uterine anomalies into six main classes: Class I – dysmorphic uterus (subclass: a – T-shaped, b – infantilis), Class II – septate uterus (subclass: a – partial, b – complete), Class III – dysfused uterus (subclass: a – partial, b – complete, Class IV – unilaterally formed uterus (subclass: a – rudimentary horn with cavity, b – rudimentary horn without cavity), Class V – aplastic/dysplastic uterus (subclass: a – rudimentary horn with cavity, b – rudimentary horn without cavity), and Class VI – unclassified malformations. Classes I–V can be further subclassified according to the coexistence of anomalies of the cervix and the vagina [5].

Most cases of unilateral kidney agenesis are asymptomatic and are diagnosed incidentally during ultrasound examinations. This condition might remain undetected until adolescence or adulthood, when complications such as hypertension or chronic kidney disease arise [2]. Prenatal diagnosis using ultrasound is possible but challenging, as it relies on the non-visualization of the kidney and may not affect the amniotic fluid volume [46]. MDA, on the other hand, is rarely detected before puberty. In most cases, OHVIRA presents as a double uterus with unilateral blind hemivagina and ipsilateral renal agenesis. HHWS does not exhibit complaints until menarche, when patients present with progressive pain due to hydrometrocolpos and hemivaginal obstruction [47]. Diagnosing MDA before puberty is important due to potential complications such as endometriosis, pelvic inflammation, fallopian tube adhesions and future fertility issues [3]. Moreover, an early identification helps in planning the follow-up care and treatment plans. Ultrasound is currently routinely used and is a reasonable initial imaging modality for evaluating both conditions. If further evaluation is warranted, MRI is used for difficult or inconclusive cases, or in cases with suspected cervical/vaginal anomalies [47,48,49]. It provides detailed images of the structures and helps in identifying associated kidney anomalies [50].

The comparison of our cases with those found in the published literature highlights several key differences and similarities. In our cases, kidney agenesis was identified before the MDA, whereas, in the literature review, simultaneous diagnoses were more common. Prenatal diagnoses were rare in the literature and were not made in either of our cases. Type III MDAs were consistently the most prevalent. While additional malformations were noted in 20% of the literature cases, our patients did not have any. Lastly, while the majority of the literature cases required urgent surgical interventions due to symptoms, only one of our cases necessitated such an intervention, with the other being asymptomatic and diagnosed incidentally.

While the co-occurrence of congenital *solitary functioning kidney* (SFK) and Müllerian duct anomalies has been documented in numerous cases and studies, screening girls with SFK for uterine and vaginal anomalies has only recently been recommended [51], but it has not been widely implemented. Management of both conditions requires a multidisciplinary approach involving pediatric nephrologists, gynecologists, and potentially other specialists. Pre-menarche patients with asymptomatic OHVIRA should undergo a regular follow-up until menarche. Surgical intervention, such as excision of the vaginal septum, is often necessary for symptomatic or post-menarche patients [3]. Post-operative long-term follow-up is required for MDA patients, especially those with Herlyn-Werner-Wunderlich syndrome, to evaluate both kidney and gynecological issues by a pediatric nephrologist and a pediatric and adolescent gynecologist [52]. Pregnancy in women with OHVIRA is categorised as high-risk and leads to an increased need for Cesarean sections.

## Conclusions

Unilateral kidney agenesis is frequently associated with Müllerian duct anomalies, emphasizing the need for a comprehensive evaluation in affected patients. An early diagnosis and management of MDA is crucial to prevent complications such as endometriosis and fertility issues. Increased clinical awareness and further research is needed to understand the underlying causes and improve screening for an early detection and better patient outcomes.
